# Soil-transmitted helminths, intestinal protozoa and *Clonorchis sinensis* infections in southeast China

**DOI:** 10.1186/s12879-021-06879-x

**Published:** 2021-11-27

**Authors:** Yan Feng, Kegen Yu, Hualiang Chen, Xuan Zhang, Qiaoyi Lu, Xiaoxiao Wang, Xueying Zhang, Linong Yao, Wei Ruan

**Affiliations:** 1 Department of Infectious Disease Prevention and Control, Zhejiang Provincial Center for Disease Control and Prevention, Hangzhou, 310051 China; 2 Department of Environmental Medicine and Public Health, Icahn School of Medicine at Mount Sinai, NewYork, 10029 USA

**Keywords:** Parasitic intestinal diseases, Soil-transmitted helminths, Protozoa infections, *Clonorchis sinensis*, Prevalence

## Abstract

**Background:**

Extensive parasitic diseases epidemiology in Zhejiang province has not been carried out since the second national survey in 2004. Therefore, dynamics in prevalence and infection pattern of the major intestinal parasites should be explored.

**Methods:**

The distribution of three parasites including soil-transmitted helminths (STH), intestinal protozoa and *C. sinensis* in Zhejiang from 2014 to 2015 were explored. Kato-Katz technique was used for STH and *C. sinensis* detection, whereas transparent adhesive paper anal swab was used for pinworm detection, and iodine smear was used for protozoa detection. A questionnaire survey on alimentary habits and sanitary behaviors was conducted in half of the studied counties.

**Results:**

This study recruited 23,552 participants: 19,935 from rural and 3617 from urban area. Overall prevalence of intestinal helminth infections was 1.80%. In this study, seven helminth species were identified including *A. duodenale*, *N. americanus*, *Trichuris trichiura, Ascaris lumbricoides*, *C. sinensis*, *Fasciolopsis buski* and pinworm. The average prevalence of STH infection was 1.71%: 1.94% in rural and 0.44% in urban area. Hookworm was the most prevalent infection at 1.58%: 1.79% in rural and 0.44% in urban area. Prevalence varied considerably in the studied counties. Prevalence was highest in Yongkang county at 10.25%. Only 2.79% of children from rural area were infected with pinworm. A proportion of 0.40% of rural participants were infected with protozoa, whereas *Endolimax nana* was the most prevalent at 0.23%. *C. sinensis* showed infection only in one man. Awareness on *C. sinensis* was 24.47% in rural and 45.96% in urban area, respectively.

**Conclusions:**

Prevalence of STH and protozoa infections declined considerably whereas *C. sinensis* infections remained few in Zhejiang province compared with the prevalence reported in previous large scale surveys (19.56% for national STH infection in 2004, 18.66% and 4.57% for provincial STH and protozoa infection, respectively in 1999). The findings of this study showed that hookworm, mainly *N. americanus* remained a parasitic threat to population health, mainly in the central and western Zhejiang. Therefore, more health education regarding fertilization and farming habits is necessary in rural areas. The awareness concerning hookworm infection should be reinforced.

**Supplementary Information:**

The online version contains supplementary material available at 10.1186/s12879-021-06879-x.

## Background

Intestinal nematode infections, also known as soil-transmitted helminths, including ascariasis, hookworm disease, and trichuriasis are a huge burden globally. These infections are associated with an estimated 5.19 million disability-adjusted life year (DALY), ranking first in the list of the estimated DALYs of Neglected Tropical Diseases (NTDs) [1]. Studies report that most cases of high-burden NTDs actually occur in Asian countries including China, India, and Indonesia [1]. Helminthiasis, protozoan infection, and clonorchiosis are most frequently reported parasitic diseases in China. However, their prevalence are likely to be underestimated since their symptoms are not clear at an early stage of infection [2–5].

Soil-transmitted helminth (STH) infections are widely distributed in tropical and subtropical areas. In addition, they are linked to poor sanitation. Therefore, STH infections are prevalent in poor countries or regions [6]. Studies reported that > 880 million children acquire STH yearly [6]. In 2010, a total of 5.3 billion people and 1.0 billion school-aged children living in areas regarded as habitats presented with at least one STH species. Furthermore, 69% of these patients were living in Asia [7]. In 2003, the prevalence of all STH species including roundworm (*Ascaris lumbricoides*), whipworm (*Trichuris trichiura*) and hookworms (*Necator americanus* and *Ancylostoma duodenale*) [8] was highest in China, followed by Southeast Asia and India [9]. However, only a few studies have explored prevalence of STH species. Studies conducted in South Asia and South East Asia reported that in 2018, roundworm was the main cause of STH at 18%, followed by whipworm at 14% and hookworm at 12% [10]. China carried out two national surveys on parasitic infections in 1992 and 2001–2004. The overall prevalence of roundworm, whipworm and hookworm in 1992 was 47.0%, 18.8%, and 17.2%, respectively [11], whereas the prevalence in 2004 was 12.72%, 4.63% and 6.12%, respectively [12]. However, these large-scale STH infection investigation has been scanty in recent years. STH infection prevalence and distribution pattern is likely to exhibit great dynamics due to current economic development as well as sanitation improvement.

Inadequate clean water, sanitation, and poor hygiene may contribute to transmission of intestinal protozoa including *cryptosporidium, Giardia intestinalis* and *Entamoeba histolytica* [13, 14]. Therefore, intestinal protozoa infections occur predominantly in developing countries.

*Cryptosporidium*, the causative agent for cryptosporidiosis, is one of the common causes of diarrhea and gastroenteritis in humans. Furthermore, it causes a great health burden to children and immunocompromised persons [15–17]. Incidences of cryptosporidiosis ranges from 1.4% to 10.4% in various countries. In addition, the rate is higher in low-income countries (5–10%) compared with the rate in developed nations (1%) [18]. *G.intestinalis* causes giardiasis, which is also a common cause of chronic diarrhea. Cases of giardiasis are mainly reported in developing countries among children. In developing countries, childhood giardiasis prevalence range is 20–30%, whereas only 2–3% prevalence is reported in the developed countries [17]. Amebiasis Amebiasis caused by *E. histolytica* affects 50 million people leading to 100,000 annual deaths globally [19]. In China, a national survey carried out in 1992 reported that the infection rates of *G. intestinalis* was 2.52% whereas that of *E. histolytica* was 0.95% [11]. However, the national survey performed between 2001 and 2004 reported a significant decrease in the prevalence. In Zhejiang province, the prevalence of *G. intestinalis* decreased from 3.85% to 1.30% whereas the prevalence of *E. histolytica* decreased from 1.5% to 0.14% compared with the previous survey [20].

Human liver fluke is also a common threat to people’s health in East Asia. *C. sinensis* is the most common human liver fluke parasite, whose infection results in adverse effects. *C. sinensis* causes severe cholangiocarcinoma, therefore, it was considered a group 1 biological carcinogen by the International Agency of Cancer Research in 2009 [21]. Moreover, due to its high prevalence, *C. sinensis* causes a huge health burden in East Asian countries including China. Approximately 15 million people were infected with *C. sinensis* globally in 2004 [22], and 85% of these infections were recorded in China [23]. Infection rate of *C. sinensis* was 0.365% [24] in 1992 and the rate increased to 0.58% [12] in the early 2000s. Two epidemic zones of *C. sinensis* were reported in China: Heilongjiang and Liaoning provinces in northern China, and Guangdong and Guangxi provinces in southern China. Zhejiang province is located in southeast China. Prevalence of *C. sinensis* was low in Zhejiang province. In the second national survey, prevalence of *C. sinensis* in Zhejiang was 0.01% [20], which was significantly lower compared with the national average (0.58%) [12]. However, parasite transmission and population composition has greatly changed rapidly in recent decades. Therefore, it is important to perform a survey to explore the distribution of *C. sinensis* in Zhejiang province [25].

The epidemiology of infections from the three parasite species introduced above (STH, intestinal protozoa, and *C. sinensis*) have not been recently explored through large-scale studies, as the last two national surveys were completed in 1992 and 2003, respectively. However, over a decade later, a high-speed development period has been observed in China and some parasitic infection rates have declined. However, infections like giardiasis persisted among the children and underprivileged communities show increased rates [18]. This study sought to explore the distribution of three different parasites in Zhejiang province. The findings of this study will contribute to eradication and prevention of intestinal parasitic infections in Zhejiang province thus reducing the health burden associated with these parasites.

## Methods

### Study sites and sampling

Zhejiang province is located in the southeast China. It is characterized by a monsoon season, temperate climate, and a mountainous landscape. Notably, 74.63% of its landscape is mountainous and hilly [26]. It is among the smallest provinces in China. An extensive field sample collection and survey was carried out in 34 counties out of the 89 counties (38.20%) in Zhejiang province from 2014 to 2015 as part of the third national parasitic infection survey. Each study county was considered a single study site, and was assigned to rural or (and) urban area. All the study sites were presented in Additional files 1 and 2. Prevalence of STH and *C. sinensis* was determined in each study site. Presence of intestinal protozoa was explored in the rural areas. Participants were included based on a multi-stage cluster sampling technique. The number of all rural participants recruited in this study was based on the national monitoring data using binomial distribution. The smallest selection unit in rural setting was a village comprising 250 participants. Then, the number of rural sites, proportionate to the county rural population size, was calculated with regard to the precious two numbers. Each county was subdivided into three or four categories based on the town-level GDP (2011 Zhejiang provincial statistic yearbook). One town was randomly selected for per each GDP level. One village was then randomly selected from the town. The number of urban participants was calculated using Poisson distribution with respect to prevalence in previous national study findings of 27 provinces in 2004 [27]. The smallest unit for the urban site was neighborhood comprising 250 participants. The number of all urban sites was determined based on the two previous numbers. The number of urban sites in each county, proportionate to the county’s urban population size, was assigned and was equivalent to the number of the studied neighborhoods. Districts were chosen randomly from each county and then neighborhoods were selected randomly from each district. In each village and neighborhood, two-hundred and fifty subjects were sampled by randomly selecting the families. All family members were requested to participate once a family was selected.

### Study population and sample collection

Permanent residents in the chosen rural sites were underwent diagnosis for STH, *C. sinensis* and intestinal protozoa infection. In addition, children aged 3–6 years old were examined for pinworm infection. Permanent residents in the urban areas were screened for both STH and *C. sinensis* infection. Demographic variables, such as sex, age, ethnicity, occupation, and education level of all participants were recorded. Approximately 30 g stool sample was collected from each participant. An additional anal swab was taken from all child participants.

### Ethics approval and consent to participate

This study was part of an Epidemiology Survey of Important Parasitic Diseases in China. It was approved by the Ethical Review Committee of National Institute of Parasitic Disease, Chinese Center of Disease Control and Prevention. Written or verbal consents were obtained from each participant. Some elderly participates, mainly in the rural areas were illiterate, therefore, to standardize the informed consent form in Zhejiang province, verbal informed consent was obtained from all the participants before sample collection and face-to-face survey. The study staff explained the content to the participants with the help of the local authorities such as the village leaders and women directors. When the villagers agreed to participate in the study, they sent their own samples themselves or those of their children.

### Sample examination

Kato-Katz technique was used to detect STH and direct faecal smears following WHO recommendation. Iodine solution was used for determination of protozoa cysts [28]. Two thick-smear slides were processed using modified Kato-Katz technique under a light microscope at 10× and 40× magnification for eggs detection in each stool sample. In addition, one slide was processed using iodine solution under light microscope at 40 magnification for detection of protozoa cysts. The average number of eggs on two Kato-Katz slides was determined when any helminth egg was detected. The findings were reported as positive or negative for the protozoa cysts. Pinworm eggs were examined under light microscope at 10× using the transparent adhesive paper anal swab technique and reported as positive or negative for pinworm eggs.

### Stool culture

Stool samples were cultured for detection of hookworms. Approximately 0.5 g of each stool sample was spread on spindly filter paper. The filter paper was then placed in a cone-shaped tube with cold boiled water at the bottom of the tube. The sample was then incubated for 4 days at 30 °C. During the incubation, water was added when water levels were low to prevent the tube from drying up. The water at the bottom was collected and examined under anatomical lens for *A. duodenale* or *N. americanus* larvae after spontaneous sedimentation. The species name and the number of larvae were recorded. A maximum of 50 samples per county were cultured.

### Infection intensity classification

The intensity of the infection was determined by eggs per gram (EPG), which was calculated through the formula: EPG = average eggs of the two slides from one sample*24. The intensity of parasites were calculated as follows, intensity of hookworm: 0 < EPG < 400, intensity = light; 400 ≤ EPG ≤ 3000, intensity = moderate; EPG > 3000, intensity = heavy; intensity of *A. lumbricoides*: 0 < EPG < 5000, intensity = light; 5000 ≤ EPG ≤ 50000, intensity = moderate; EPG > 50000, intensity = heavy; intensity of *T. trichiura*: 0 < EPG < 1000, intensity = light; 1000 ≤ EPG ≤ 10000, intensity = moderate; EPG > 10000, intensity = heavy.

### Quality control

Two trained laboratory staff examined the two Kato-Katz slides for each stool sample. An experienced parasitologist then re-checked the slides in case the two trained staff reported inconsistent findings. Afterwards, an experienced parasitologist re-observed the iodine slide when cysts of protozoa were detected. Lastly, 10% of the positive samples and 5% of the negative samples were then examined by the provincial experienced parasitologists.

### Questionnaire survey

Questionnaires (Additional files 3 and 4) were developed for this study whereby surveys were carried out in 17 counties randomly selected from the 34 study counties. A total of 60 subjects including those under stool examination in each study unit were filled the questionnaires based on alimentary habits. Notably, sanitary behaviors were recorded for the rural areas.

### Statistical analysis

Questionnaire data and laboratory examination findings were recorded in independently by two research staffs using Epi-Info 3.5.4 (CDC, GA, USA). Data were then compared to avoid discrepancies, logical errors and missing values. Statistical analysis was conducted performed using SPSS 24 software (SPSS Inc., IL, USA). Pearson’s chi-squared test (χ2) was used to compare the crude associations between binary and independent variables. Significance level for all the tests was set at < 5% (*P* < 0.05).

## Results

### Characteristics of study sites

Seventy-eight villages in the rural area and 14 urban neighborhoods were chosen for this study. Landscapes of selected villages (rural study sites) were: mountainous (28, 35.90%), hilly (25, 32.05%), plain (20, 25.64%) and others (5, 6.41%). On the other hand, the urban study sites were mainly characterized by plain landscape (6, 42.86%). Most villages (76, 90.48%) had access to tap water. Most villages did not report the habit of eating raw meat, however, one village in Wenzhou city ate raw freshwater fish and shrimp. Local drinking and eating habits are presented in Table 1.

**Table 1:** Local drinking and food habits in rural area

Habits	The NO. (%) of villages	
	N	%
Major drinking water source		
Tap water	76	90.48
Well water	3	3.57
Pond water	2	2.38
Spring water	3	3.57
Raw meat diet		
Yes	0	0
No	84	100%
Raw freshwater fish & shrimp diet		
Yes	1	1.19
No	83	98.81
Raw vegetable diet		
Yes	50	59.52
No	34	40.48
Air-dried meat and fish		
Yes	28	33.33
No	56	66.67

### Characteristics of participants

The characteristics of the study population were presented in Table 2. A total of 23,552 participants were included in this study. Among them, 19,935 participants were from the rural whereas 3617 participants were from the urban areas. Number of the female participants (10487) was significantly higher compared with that of the male participants (9448) in both the rural and urban sites. The average age of the rural and urban participants was 53 and 49, respectively. Ethnic Han accounted for 98.50% of the study population, whereas Ethnic She, which was a minority cluster in Zhejiang province accounted for 1.44% of participants. Type of occupations varied among of participants in both rural and urban areas. Although farming was the predominant occupation in both rural and urban areas, farming rate was significantly higher in rural area compared with that of urban areas. Proportions of the participants who had completed primary school and middle school were similar in rural and urban areas. On the other hand, the proportion of participants with high school, college and above education degrees were significantly higher in urban compared with that in rural areas.

**Table 2:** Characteristics of participants in rural area and urban area

	Rural area		Urban area		χ^2^/χ_c_^2^	*p*	Total	
	N	(%)	N	(%)			N	(%)
Age								
1–17	2008	10.07	424	11.72	9.00	< 0.05	2432	10.33
18–44	3967	19.90	836	23.11	19.473	< 0.05	4803	20.39
45–60	7012	35.17	1062	29.36	3.24	0.07	8074	34.28
> 60	6948	34.85	1295	35.80	1.21	0.27	8243	35.00
Sex								
Female	10487	52.61	1968	54.41	4.00	< 0.05	12,455	52.88
Male	9448	47.39	1649	45.59			11097	47.12
Ethnicity								
Ethnic Han	19619	98.41	3580	98.98	6.55	0.01	23199	98.50
Ethnic She	303	1.52	36	1.00	5.94	0.02	339	1.44
Others	13	0.07	1	0.03	0.88^a^	0.35	14	0.06
Job								
Farmer	13922	69.84	869	24.03	2.8 × 10^3^	< 0.05	14791	62.80
Worker	1346	6.75	420	11.61	104.25	< 0.05	1766	7.50
Housewife	1522	7.63	339	9.37	12.70	< 0.05	1861	7.90
Student	1250	6.27	320	8.85	32.67	< 0.05	1570	6.67
Preschoolers	856	4.29	147	4.06	0.40	0.53	1003	4.26
Officer	342	1.72	294	8.13	479.16	< 0.05	636	2.70
Retired	204	1.02	825	22.81	3.5 × 10^3^	< 0.05	1029	4.37
Others	493	2.47	403	11.14	628.66	< 0.05	896	3.80
Education level								
Illiteracy	4228	21.21	482	13.33	118.91	< 0.05	4710	20.00
Primary school	8555	42.91	1204	33.29	116.93	< 0.05	9759	41.44
Middle school	5218	26.18	1099	30.38	27.64	< 0.05	6317	26.82
High school	1357	6.81	470	12.99	163.78	< 0.05	1827	7.76
College and above	577	2.89	362	10.01	404.75	< 0.05	939	3.99
Total	19935	100	3617	100			23552	100

^a^If theoretical frequency (T) in the table was between 1 and 5, correction for continuity (Yate’s correction) was performed with χ_c_^2^

### Prevalence of helminth infection

Overall prevalence of intestinal helminth was 1.80% in Zhejiang province. Seven helminth species were detected including: *A. duodenale*, *N. americanus*, *T. trichiura*, *A. lumbricoides*, *C. sinensis*, *F. buski* and pinworm. Prevalence of STH infection in Zhejiang province was 1.71%. STH prevalence was significantly higher in rural areas (1.94%) compared with that in urban areas (0.44%) (*P* < 0.05) (Table 3). Hookworm was the most prevalent parasite (1.58%)*,* followed by *T. trichiura* (0.08%) and *A. lumbricoides* (0.06%). The prevalence of the hookworm was higher in the rural areas (1.79%) compared with that the urban areas (0.44%) (*P* < 0.05). *T. trichiura* and *A. lumbricoides* were only detected in samples from rural areas. Most of the participants presenting with parasite infection exhibited only one type of infection. Three participants (0.01%) exhibited co-infection with of *T. trichiura* infection and *A. lumbricoides*. No participant showed more than two types of infections. Analysis of hookworm infection showed a higher prevalence in counties located in the central and western Zhejiang compared with other study counties. Yongkang, a county located in central Zhejiang, for instance exhibited the highest hookworm prevalence (10.25%), followed by Kaihua (6.80%) and Jiande County (6.57%). Hookworm infection was not detected in ten counties (29.41%). Most hookworm infections (70.24%) including *T.trichiura* (100%), and *A. lumbricoides* (92.31%) were of light intensity (Table 4). Male (60.59%), old (> 60) participants and farmers exhibited higher prevalence of hookworm infections (Table 5). Out of 357 hookworm positive stool samples, 330 (92.44%, 330/357) samples in rural areas were cultured, and larvae were detected in 73.94% (244/330) of the samples. *N. americanus* was the predominant (75.00%, 183/244) species in Zhejiang province (Table 6). The average number of hookworm eggs on larvae negative samples’ slides was 6.19 ± 10.77 (95% CI 3.88–8.50). Although the maximal number of hookworm eggs was high (60), most samples (62.79%) showed less than 3 eggs per slide.

**Table 3:** Prevalence of helminth infection

Parasite	The NO. and prevalence of infection							
	Rural area		Urban area		χ^2^/χ_c_^2^	*p*	Total	
	N	%	N	%			N	%
Any intestinal helminth	406	2.04	18	0.50			424	1.80
STH	387	1.94	16	0.44	40.90	< 0.05	403	1.71
Hookworm	357	1.79	16	0.44	35.72	< 0.05	373	1.58
*T. trichiura*	20	0.10	0	0	6.67^a^	0.01	20	0.08
*A. lumbricoides*	13	0.07	0	0	4.34^a^	0.04	13	0.06
Fertilized egg	8	0.04	0	0	2.67^a^	0.10	8	0.03
Unfertilized egg	6	0.03	0	0	/	0.60^b^	6	0.03
*T. trichiura* & *A. lumbricoides*	3	0.02	0	0	/	1.00^b^	3	0.01
*C. sinensis*	1	0.01	0	0	/	1.00^b^	1	0.004
*Fasciolopsis buski*	0	0	2	0.06	/	0.02^b^	2	0.01
Pinworm (anal swab)	18	2.79	NA	NA	NA	NA	18	2.79

NA, not available

^a^If theoretical frequency (T) in the table was between 1 and 5, correction for continuity (Yate’s correction) was performed with χ_c_^2^

^b^If T in the table was less than 1, exact probability method was used with only Fisher’s exact *P*

**Table 4:** Infection intensity of STH

Intensity	The NO. of Hookworm		Total N (%)	The NO. of *A. lumbricoides*		Total N (%)	The NO. of *T. trichiura*		Total N (%)
	Rural area	Urban area		Rural area	Urban area		Rural area	Urban area	
Light	251	11	262 (70.24)	12	0	12 (92.31)	20	0	20 (100)
Moderate	88	4	92 (24.66)	1	0	1 (7.69)	0	0	0 (0)
Heavy	18	1	19 (5.09)	0	0	0 (0)	0	0	0 (0)
Total	357	16	373	13	0	13	20	0	20

**Table 5:** Age and job distribution of hookworm positive participants

Characteristics	Sex (n)		Total (n)	NO. of subjects	Prevalence (%)
	Male	Female			
Age					
1–17	5	2	7	2432	0.29
18–44	4	10	14	4803	0.29
45–60	45	34	79	8074	0.98
> 60	172	101	273	8243	3.31
Job					
Farmer	197	124	321	14791	2.17
Worker	18	4	22	1766	1.25
Housewife	0	16	16	1861	0.86
Student	3	2	5	1570	0.32
Preschoolers	2	0	2	1003	0.20
Retired	5	0	5	1029	0.49
Others	1	1	2	1532	0.13
Total	226	147	373	23552	1.58

**Table 6:** Coproculture result of hookworm positive stool sample in rural areas

Coproculture result	The NO. (%)of fetal sample
Larvae positive	244 (73.94%)
*N. americanus*	183 (55.45%)
*A. duodenale*	58 (17.58%)
*A. duodenale* and *N. americanus*	3 (0.91%)
Larvae negative	86 (26.06%)
Total^a^	330 (100%)

^a^Not each positive stool was cultured in each study county

Out of 646 children tested for pinworm in rural areas using transparent adhesive paper anal swab, only 2.79% children tested positive for pinworm infection. Pinworm infection rate for boys (9, 2.50%) was not significant different from that of girls (9, 3.18%) (χ^2^ = 0.29, *P* = 0.59). Among the children between three to six years old who were tested, 6 year-old children showed the significantly higher infection rate (3.85%, 8/208), whereas the 5 year-old children had significantly lower infection rate (0.62%, 1/162) (*P* = 0.08).

### Prevalence of protozoa infection

Nine protozoa species were detected in the rural areas. Analysis showed that 0.40% of the rural participants were infected with protozoa. Notably, *Endolimax nana* was the most prevalent (0.23%) species, followed by *Entamoeba histolytica* (0.05%) and *Entamoeba hartmani* (0.04%) (Table 7). In addition, *Entamoeba coli, Entamoeba polecki*, *Giardia intestinalis*, *Trichomonas hominis*, *lodamoeba butschli*, *Blastocystis hominis* were detected, however, their prevalence was low (Table 7). The positive cases were three participants from various villages with multiple hookworm and protozoa infections.

**Table 7:** Prevalence of protozoa infection

Protozoa	The NO. and prevalence	
	N	%
*Endolimax nana*	46	0.23
*Entamoeba histolytica*	9	0.05
*Entamoeba hartmani*	8	0.04
*Entamoeba coli*	4	0.02
*Trichomonas hominis*	4	0.02
*Entamoeba polecki*	3	0.02
*Giardia intestinalis*	3	0.02
*lodamoeba butschli*	1	0.01
*Blastocystis hominis*	1	0.01
Total	79	0.40

### Prevalence of *C. sinensis*

*Clonorchis sinensis* was detected only in one man living in the rural area. On the other hand, participants from the urban area tested negative for *C. sinensis* infection. Clonorchiosis exhibited very low infection rate in Zhejiang province.

### Sanitary behaviors and alimentary habits

A total of 2485 participants (12.46% of rural participants) completed the questionnaire survey on the behaviors related to STH infection in rural study sites. Most of the participants (89.14%) exhibited good personal hygiene including washing hands before eating or after using the toilet. However, 12.52% of the people in rural areas consumed water before boiling. In addition, 21.13% of the participants used human stools as crop fertilizers whereas 22.78% participants did not wear shoes when farming. These behaviors may increase the risk of STH infection.

A total of 2485 participants from rural and 483 participants from urban areas participated in the survey on alimentary habits. 12.60% of all the participants completed the survey on knowledge, risky behaviors, and alimentary habits related to *C. sinensis* infection. The number of participants from the rural areas who had knowledge about transmission route of *C. sinensis* and its effects on health was significantly different from the number of urban participants (Table 8). The proportion of the participants who used different cutting boards for raw and cooked food was not significantly different between the rural and urban areas. A small proportion of participants (14.00% in rural area and 9.32% in urban area) ate raw freshwater fish and shrimp. Most of the participants took medicine after *C. sinensis* infection (Table 8).

**Table 8:** Knowledge, infection risky behaviors and alimentary habits related with *C. sinensis*

Investigation	Rural area N (%)	Urban area N (%)	χ^2^	*p*
Know of *C. sinensis*	608 (24.47)	222 (45.96)	92.76	< 0.05
Know of *C. sinensis* transmission route	357 (14.37)	123 (25.47)	36.75	< 0.05
Know of *C. sinensis* infection damage to the health	432 (17.38)	127 (26.29)	21.00	< 0.05
Separate raw and cooked cutting board	832 (33.48)	175 (36.23)	1.37	0.24
Eating raw freshwater fish and shrimp	348 (14.00)	45 (9.32)	7.73	0.005
Would like to try raw freshwater fish and shrimp even knowing the infection risk	182(7.32)	59 (12.22)	12.97	< 0.05
Willing to buy anthelmintic if knowing infected by *C. sinensis*	2332 (93.84)	457 (94.62)	0.43	0.51

## Discussion

More than a third of the counties in Zhejiang province were included in this study. Counties included in this study had access to clean water, fairly hygienic conditions and had no special eating habits.

Analysis showed that women were more willing to participate in the survey compared with men in both rural and urban areas. The average age of rural participants was higher compared to that of urban areas. This may be because most old people reside the rural areas whereas most young people moved to the urban areas in search of job opportunities. Urban areas included in this study were mainly previous suburban areas which were converted due to increased urbanization. The most common occupation among urban and rural participants was farming. This may explain why low-level education rate was similar among rural and urban participants. However, higher education (high school and above) was more accessible for urban residents. This observation explained the difference in number of participants who had high school and higher education degrees in urban and rural areas.

Prevalence of the intestinal parasitic infections in Zhejiang province decreased dramatically from 22.84% [20] in the second national survey (2004) to 2.12% observed in this study. Overall prevalence varied between the provinces in China as follows: 15.5% in Sichuan province (southwest China) [29], 0.18% in Jiangsu province (southeast China) [30], and 2.5% in Heilongjiang province (north China) [31]. The overall prevalence of STH infection was much higher in our study compared with 59.9% in Malaysia [32], 39% in India [33], 9.6% in southern Thailand, and 76.8%, 31.7% and 25.0% for hookworm, *A. lumbricoides* and *T. trichiura* in southern Lao People’s Democratic Republic. However, these comparisons have limitations, since the study in Malaysia targeted populations living in a jungle environment whereas the study in India targeted only rural participants. Prevalence of all STH infections was significantly higher in rural areas compared with that in urban areas. Causes of STH infection showed significant changes in Zhejiang since the second national survey [20]. A total of 17 species of parasites were detected in Zhejiang during the second national survey [20] whereas 16 species of parasites (7 species of helminth and 9 protozoa) were detected in this study. Although the number of the species recorded in the two surveys were similar, the prevalence for the two study periods exhibited great variations. Prevalence decreased dramatically from 6.84% to 0.055% for *A. lumbricoides*, 3.64% to 0.085% for *T. trichiura*, 8.18% to 1.584% for hookworm, 24.42% to 2.79% for pinworm, 0.14% to 0.045% for *Entamoeba histolytica*, and 1.00% to 0.015% for *Giardia intestinalis* [20]. The major cause of STH infection also changed from multiple helminth species to hookworm. In addition, prevalence of hookworm was higher compared with that of helminth species. Therefore, *A. lumbricoides* and *T. trichiura* were not the leading cause of helminth infection in Zhejiang province contrary to findings from second national survey. Pinworm infection was effectively controlled among children. The considerable reduction in prevalence is attributable to the rapid economic development [34], increased access to tap water and sanitary toilets, and improved personal hygiene habits including washing hands. Reports from the provincial statistical yearbook showed that the per capita income increased by 452.4% for urban residents and 486.7% for rural residents. Nationwide accessibility to tap water in the rural areas was 80% in 2017 [35]. In addition, rural tap water supply rate would stabilise above 99% from 2018 to 2022 in Zhejiang [36]. A meta-analysis study reported that use of piped water correlated with decreased probability of *A. lumbricoides* infection and *T. trichiura* infections. However, there was scanty evidence for correlation of water availability and hookworm infection [37]. In addition to tap water, nationwide accessibility to sanitary toilet showed a rapid increase from 20.9% in 1996 to 78.43% in 2015 [38]. In Zhejiang, coverage rate of sanitary toilet reached 94.60% households by 2011 [39]. However, in some rural areas, villagers, mainly the elderly, were not willing to use modern sanitary toilet and would use fresh stool as fertilizers. Furthermore, barefooted farming increased their risk to hookworm infection. In the rural areas, men were the main labor force in the field. Higher prevalence of hookworm in male, and participants who were farmers can be attributed to these habits and customs. Hookworm parasite caused the highest number of infections among Zhejiang people. Prevalence of hookworm was inversely proportional to the latitude. In South Asia and South East Asia, hookworm (12%) was the third most prevalent type of STH. Countrywide prevalence of hookworm was highest in Laos (30%, 17–48%) followed by Vietnam (29%, 14–52%) and Cambodia (28%, 18–42%) [10]. *A. duodenale* and *N. americanus* were both endemic in Zhejiang province, however, *N. americanus* was the predominant species. In the neighboring Jiangxi province, which is located to the southwest of Zhejiang, *N. americanus* exhibited high prevalence (80.41%) [40]. In Thailand, *N. americanus* was the main hookworm identified in northeast region, however, *A. duodenale* has also been reported in this region, whereas only *N. americanus* was reported in the southern region [41]. In the first national survey, a higher prevalence of *N. americanus* was reported in the southern region, whereas *A. duodenale* was predominant in the northern region [11].

The last national protozoan infection survey was carried out in the first national parasitic survey in 1998–1999 which reported a prevalence of 10.32% [24]. In the second national parasitic survey in 2004, protozoa were not included in the detection target. Provincial survey in 1999 reported a prevalence of 4.57% (717/15698) of protozoa infections and a prevalence of 0.99% of *Entamoeba coli* which was the predominant protozoan [20]. The prevalence declined sharply to 0.40% compared with prevalence from previous surveys, whereas the predominant protozoan in this study was *Endolimax nana*.

Questionnaire survey concerning *C. sinensis* revealed that although urban participants had more knowledge regarding Clonorchiosis or *C. sinensis* compared with rural participants, the overall awareness rate in urban participants was less than a half the included participants. More than a half of the participants reported that they would like to eat raw freshwater fish and shrimp even though they knew that these were the transmission routes of the parasites. A total of 9.32% urban participants ate raw freshwater fish and shrimp, and the rate increased to 12.22% among participants though they knew the risk of *C. ssinensis* infection. However, the situation was opposite among the rural participants.

This study had a few limitations. Firstly, although Kato–Katz technique for STH detection and direct fecal smears with iodine for cysts of protozoa are recommend by WHO in the field, the sensitivity of these two methods was low. Low sensitivity for hookworm detection is attributed rapid degeneration of delicate hookworm eggs with time [42, 43]. In this study, eggs were examined within 2 h after slides were properly prepared to avoid rapid degeneration of the hookworm. Two trained laboratory staff analyzed two slides for each sample to improve sensitivity. Secondly, cluster sampling was used in this study, which has more convenient implementation and lowers the cost of the study, however, it results in less representation of samples.

## Conclusion

The findings of this study show that the prevalence of STH and protozoa infections has been decreased over time. In addition, *C. sinensis* infection was not prevalent in Zhejiang. However, hookworm, particularly *N. americanus* was a parasitic threat to population health, especially in some counties in the central and western Zhejiang. Health education should carry out on use of organic fertilizers and farming habits. Moreover, the awareness concerning hookworm infections should be reinforced.

## Supplementary Information

**Additional file 1:** Study sites in rural area.

**Additional file 2:** Study sites in urban area.

**Additional file 3:** Knowledge and Behavior of Soil-transmitted helminth and *Clonorchis sinensis* Questionnaire.

**Additional file 4:** Knowledge and Behavior of *Clonorchis sinensis* Questionnaire.

## Data Availability

The datasets analyzed during the current study are not publicly available due to data sharing need the approval of affiliations but are available from the corresponding author on reasonable request.

## Abbreviations

STH: Soil-transmitted helminthiasis
NTDs: Neglected Tropical Diseases
GDP: Per capita net income
