# Supplementary material for: Soil-transmitted helminths, intestinal protozoa and Clonorchis sinensis infections in southeast China
Source: BMC Infect Dis. 2021 Nov 27;21:1195. doi: 10.1186/s12879-021-06879-x (PMC8626871; doi:10.1186/s12879-021-06879-x)
Supplement: Supplementary file 3 — Additional file 3: Knowledge and Behavior of Soil-transmitted helminth and Clonorchis sinensis Questionnaire. [file 12879_2021_6879_MOESM3_ESM.docx]

Knowledge and Behavior of Soil-transmitted helminth and *Clonorchis sinensis* Questionnaire

Province City County Town Village

**Site NO. Case NO.**

**Part 1 General information**

Q1.Name：

Q2.Sex：①male；②female

Q3.Age：

**Part 2 Soil-transmitted helminth disease section**

**A．Knowledge (Investigator judged by the reference answer in brackets)**

Q1. Have you heard of roundworm, whipworm or hookworm? ①Yes ②No (Answer Q7)

Q2. How is roundworm, whipworm infected? (eg. food is not clean, eat worm eggs by mistake) ①know ②don't know

Q3. How is hookworm infected? (Infected through the skin or oral transmitted) ①know ②don't know

Q4. If there are worms in human body, is there any harm to people? (Yes, abdominal pain, diarrhea, anemia, malnutrition) ①know ②don't know

Q5. What can be done to prevent roundworm, whipworm, and hookworm infection? (Wash hands frequently, do not drink raw water, ware shoes when farming) ①know ②don't know

**B. Prevention behavior**

Q7. Do you wash your hands before eating and after using the toilet? ①Yes ②No

Q8. Do you drink unboiled water? ①Yes ②No

Q9. Do you use human stools to fertilize crops? ①Yes ②No

Q10. Do you work in the field barefoot? ①Yes ②No

**C. Attitude**

Q 11. Are you willing to buy deworming medicine if you are infected? ①Yes ②No

Q 12. What would you do if your behavior is under risk of parasite infection? ①do not change the behavior ②buy deworming medicine ③change those risky behavior

**Part 3 clonorchis sinensis disease section**

**A．Knowledge (Investigator judged by the reference answer in brackets)**

Q1. Have you heard of clonorchis sinensis (liver fluke)? ①Yes ②No (Answer Q5)

Q2. How is liver fluke infected? (eat raw or undercooked freshwater fish) ①know ②don't know

Q3. Is liver fluke harmful to human body? (Cause abdominal pain, diarrhea, fatigue, cause cholecystitis, gallstones and other diseases of the hepatobiliary system) ①know ②don't know

Q4. How to prevent liver fluke infection? (do not eat raw freshwater fish or shrimp) ①know ②don't know

**B. Prevention behavior**

Q5. Do you like to eat raw or undercooked freshwater fish or shrimp?

①Yes (Answer Q6, 8, 9) ②No (Answer Q6, 7, 9)

Q6. Do you separate raw and cooked cutting boards? ①Yes ②No

**C. Attitude**

Q7. Would you like to try the delicious Sashimi even though they are under risk of liver fluke infection? ①Yes ②No

Q8. Are you willing to buy deworming medicine if you are infected by liver fluke infection? ①Yes ②No

Q9. Would you like to continue to eat raw or undercooked freshwater fish and shrimp after cure of clonorchiasis? ①Yes ②No

Investigator: Date:
