# Supplementary material for: Soil-transmitted helminths, intestinal protozoa and Clonorchis sinensis infections in southeast China
Source: BMC Infect Dis. 2021 Nov 27;21:1195. doi: 10.1186/s12879-021-06879-x (PMC8626871; doi:10.1186/s12879-021-06879-x)
Supplement: Supplementary file 4 — Additional file 4: Knowledge and Behavior of Clonorchis sinensis Questionnaire. [file 12879_2021_6879_MOESM4_ESM.docx]

Knowledge and Behavior of *Clonorchis sinensis* Questionnaire

Province City County Subdistrict Committee

Site NO. Case NO.

**Part 1 General information**

Q1.Name：

Q2.Sex：①male；②female

Q3.Age：

**Part 2 Knowledge (Investigator judged by the reference answer in brackets)**

Q1. Have you heard of *clonorchis sinensis* (liver fluke)? ①Yes ②No (Answer Q5)

Q2. How is liver fluke infected? (eat raw or undercooked freshwater fish) ①know ②don't know

Q3. Is liver fluke harmful to human body? (Cause abdominal pain, diarrhea, fatigue, cause cholecystitis, gallstones and other diseases of the hepatobiliary system) ①know ②don't know

Q4. How to prevent liver fluke infection? (do not eat raw freshwater fish or shrimp) ①know ②don't know

**Part 3 Prevention behavior**

Q5. Do you like to eat raw or undercooked freshwater fish or shrimp?

Yes (Answer Q6, 8, 9) ②No (Answer Q6, 7, 9)

Q6. Do you separate raw and cooked cutting boards? ①Yes ②No

**Part 4. Attitude**

Q7. Would you like to try the delicious Sashimi even though they are under risk of liver fluke infection? ①Yes ②No

Q8. Are you willing to buy deworming medicine if you are infected by liver fluke infection? ①Yes ②No

Q9. Would you like to continue to eat raw or undercooked freshwater fish and shrimp after cure of clonorchiasis? ①Yes ②No

Investigator: Date:
